# Facile Fabrication of Hybrid Carbon Nanotube Sensors by Laser Direct Transfer

**DOI:** 10.3390/nano11102604

**Published:** 2021-10-03

**Authors:** Anca F. Bonciu, Mihaela Filipescu, Stefan I. Voicu, Thomas Lippert, Alexandra Palla-Papavlu

**Affiliations:** 1 Lasers Department, National Institute for Lasers, Plasma and Radiation Physics, Atomistilor 409, 077125 Magurele, Romania; anca.bonciu@inflpr.ro (A.F.B.); mihaela.filipescu@inflpr.ro (M.F.); 2 Faculty of Physics, University of Bucharest, Atomistilor 409, 077125 Magurele, Romania; 3 Department of Analytical and Environmental Chemistry, University Politechnica of Bucharest, 1-7 Gh. Polizu Str., 011061 Bucharest, Romania; svoicu@gmail.com; 4 Advanced Polymer Materials Group, University Politehnica of Bucharest, 1-7 Gh. Polizu Str., 011061 Bucharest, Romania; 5 Laboratory of Inorganic Chemistry, Department of Chemistry and Applied Biosciences, ETH Zurich, 8093 Zurich, Switzerland; thomas.lippert@psi.ch; 6 Laboratory of Multiscale Materials Experiments, Paul Scherrer Institute, 5232 Villigen, Switzerland

**Keywords:** gas sensor, ammonia detection, hybrid, LIFT, laser, nanocomposite, SWCNT, SnO_2_

## Abstract

Ammonia is one of the most frequently produced chemicals in the world, and thus, reliable measurements of different NH_3_ concentrations are critical for a variety of industries, among which are the agricultural and healthcare sectors. The currently available technologies for the detection of NH_3_ provide accurate identification; however, they are limited by size, portability, and fabrication cost. Therefore, in this work, we report the laser-induced forward transfer (LIFT) of single-walled carbon nanotubes (SWCNTs) decorated with tin oxide nanoparticles (SnO_2_ NPs), which act as sensitive materials in chemiresistive NH_3_ sensors. We demonstrate that the LIFT-fabricated sensors can detect NH_3_ at room temperature and have a response time of 13 s (for 25 ppm NH_3_). In addition, the laser-fabricated sensors are fully reversible when exposed to multiple cycles of NH_3_ and have an excellent theoretical limit of detection of 24 ppt.

## 1. Introduction

Ammonia (NH_3_) is one of the most frequently produced industrial chemicals in the United States and China [1]. NH_3_ is extensively used in various industrial applications, i.e., production of fertilizers, plastics, dyes, etc. Exposure to high concentrations of ammonia in air causes bronchiolar and alveolar edema, while the inhalation of lower concentrations can cause coughing, nose, and throat irritation [2]. According to the National Institute for Occupational Safety and Health (NIOSH), the acceptable exposure limit is 25 ppm for 8 h and 35 ppm for 15 min exposure. 

Therefore, detection of ammonia requires efficient devices with several key characteristics, i.e., sensitivity, the minimum concentration of target gases they can detect, response speed, reversibility, energy consumption, and fabrication costs [3]. 

For the last 50 years, the field of sensors has been dominated by metal oxide semiconductor sensors based on SnO_2_, In_2_O_3_, ZnO_2_, WO_3_, etc. [4]. This type of sensor has numerous advantages in terms of raw material costs, high sensitivity, etc. However, their main disadvantage is that they have to be operated at higher temperatures, (e.g., SnO_2_ at 500 °C), which makes difficult their application on flexible substrates [5]. 

The field of sensors is far from developed, and since the pioneering work of Dai et al. [6], in which the first examples of single-walled carbon nanotube (SWCNT) chemical sensors were reported, sensors based on CNT have gained great interest. Resistive SWCNT-based sensors are low-power devices that are ideal for direct electrical detection [7]. SWCNTs have been proven effective for the detection of common organic vapors, [8] oxidizing vapors such as Cl_2_ or NO_2_, [9] and even trinitrotoluene [10] and chemical warfare agents [11]. The main drawbacks for the application of SWCNT in sensors are the high price and the necessity to find techniques to deposit them on the active area of a resistive sensor with high enough conductivity [12]. 

Although there are numerous examples of sensors based on pristine CNTs, different reports available in the literature [13] associate an increased sensor response and sensitivity toward different analytes to residual metal catalysts or particles from the production process. Thus, hybrid carbon-based sensing materials can be developed by intentionally decorating or doping carbon nanotubes with metal nanoparticles in order to produce a stronger and specific response toward the analytes of interest [14]. 

Therefore, in this work, we focused on an easy and straightforward sensor device fabrication strategy that relies on laser printing of the sensing materials by laser-induced forward transfer (LIFT). LIFT is a solvent-free and versatile fabrication method, which has already been applied for device fabrication [15,16]. The basic principle of LIFT relies on shaping and projecting a laser beam onto a donor substrate coated with the material to be transferred. The donor substrate coated with the material to be transferred is then placed parallel and at a controllable distance to a receiving substrate, and the entire system is moved with respect to the fixed laser beam so that it is possible to “write” arbitrary patterns. Laser transfer of material takes place when a single laser pulse is fired. From the large donor substrate, a selective transfer of only a small part defined by the laser spot can be achieved, without any additional photolithography step. Using this configuration, the laser interacts with the material to be transferred and only insensitive materials such as metals or ceramics can be transferred. In order to transfer materials that can be damaged by the laser beam (generally proteins or polymers), the LIFT process needs to be improved by adding an intermediate layer between the donor substrate and the material to be transferred with the role to protect the material to be transferred from the applied laser light and generated heat. 

Few examples of carbon nanotube transfers by LIFT have been reported previously; for example, in [16], we have proved that it is possible to transfer SWCNT by LIFT with the goal of fabricating chemiresistive sensors that detect ammonia in the ppm range. Furthermore, in [17], the authors demonstrated the possibility to print polymer-multiwalled carbon nanotube composites by LIFT, while in [18], the authors demonstrated the transfer of SWCNTs and SWCNTs embedded in a polymer matrix by using a blister to mechanically deform the donor layer. Moreover, in [19], the authors presented the possibility to LIFT and decorate multiwalled carbon nanotubes with gold–palladium nanoparticles and demonstrated the adsorption characteristics toward hydrogen. 

Therefore, in this study, we focused on transferring hybrid SWCNT, i.e., decorated with tin oxide nanoparticles (SWCNT@SnO_2_) with the purpose of obtaining improved and reproducible resistive sensors. We report, for the first time, the fabrication of resistive sensors by laser-induced forward transfer of hybrid nanocomposites, i.e., SWCNT@SnO_2_ with an excellent detection limit toward ammonia in the low ppt range. 

## 2. Materials and Methods

### 2.1. Preparation of the Materials to Be Transferred—Donor Fabrication

The materials to be transferred by laser-induced forward transfer were single-walled carbon nanotubes (SWCNT, HiPco) purchased from Nanointegris, and hybrid nanocomposites based on SWCNT and SnO_2_ nanoparticles were purchased from Alfa Aesar. We chose SWCNT over MWCNT, taking into account gas–nanostructure interactions, and the work of Picaud et al. [20], in which the authors have reported that for the same amount of NH_3_ molecules adsorbed on the two types of carbon nanotubes, the SWCNTs appear to have the best sensitivity to NH_3_.

The SWCNT@SnO_2_ dispersions were realized at a 1:14 SWCNT: SnO_2_ ratio. Briefly, the SWCNTs were used as received, without any additional purification step. Dispersions of SWCNTs in water were realized by suspending 10 mg CNT powder in 15 mL water containing 100 mg Triton X. The SnO_2_ nanoparticles used in this work had a nominal particle size of 10–15 nm and were dispersed in a colloidal solution at a concentration of 14 wt.% in water. The dispersion was a bath sonicated for 40 min.

The hybrid SWCNT@SnO_2_ nanocomposites were prepared by dispersing the HiPco SWCNT in the SnO_2_ NP solutions by ultrasonic vibration for about 2 h to obtain the well-mixed suspensions. 

**Figure 1:**
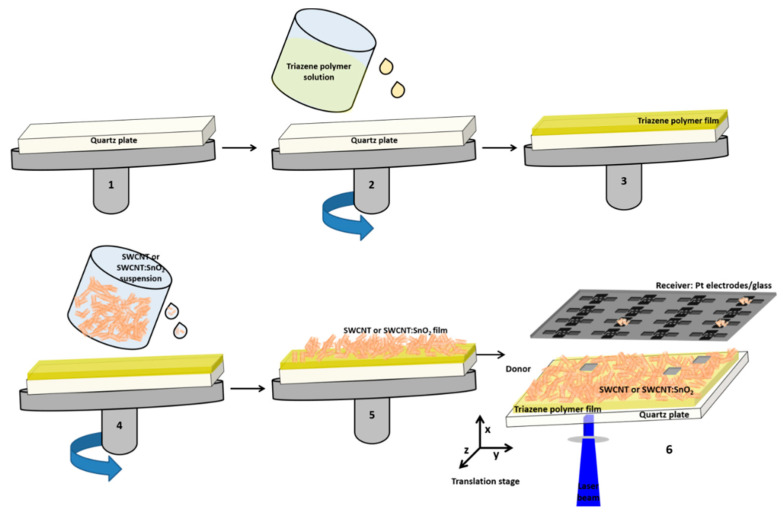
Scheme of the LIFT setup: step 1–5 fabrication of the donor substrate by spin coating, and step 6 transfer by LIFT of the donor material (SWCNT@SnO_2_) onto the receiver substrate (glass plate with Pt electrodes). Step 1: The donor plate was placed in the spin coater. Step 2 and 3: The quartz plate was coated with a thin film of the triazene polymer (TP). Step 4 and 5: The SWCNT@SnO_2_ suspension was spin-coated onto the TP/quart plate. Step 6: The donor and receiver were placed onto an *xyz* translation stage. The laser beam impinges through the transparent quartz plate, vaporized the TP, which mechanically pushed forward a small portion of the SWCNT@SnO_2_ film onto the glass plate with Pt electrodes.

The preparation of the TP layer, the SWCNT, and hybrid SWCNT@SnO_2_ layers was carried out following the procedure described schematically in Figure 1.

In order to prepare the donor films for the LIFT experiments, a preliminary step of coating fused silica plates with a photosensitive polymer, i.e., a triazene polymer (TP), was carried out. The specific triazene polymer was synthesized, following the procedure published previously [21]. The triazene polymer was deposited by spin coating from a 2% wt solution of chlorobenzene and cyclohexanone (1:1 *w*/*w*). The TP-containing solution was dispensed onto the substrate through a 0.45 μm filter. Spinning was carried out for 60 s at a speed of 2000 rpm with a ramp of 1000 rpm/s. The films were dried after deposition for 2 h at 50 °C. Films with a thickness of 150 nm were obtained with this procedure.

The SWCNT and SWCNT@SnO_2_ layers were fabricated by spin coating the SWCNT and SWCNT@SnO_2_ dispersions (different experiments) onto TP-coated fused silica plates under different conditions, i.e., rotation speed 1500–2500, a ramp of 1000–2000 rpm, and spin-coating duration of 30 and 60 s. Once the final donor layers were obtained, a post-heating step (60 °C for 4 h) was applied to remove the residual solvent from the film.

### 2.2. LIFT

The LIFT setup used in this work has been detailed in our previous works [5,16] and it consists of a pulsed XeCl laser (308 nm emission wavelength, 30 ns pulse length, 1 Hz repetition rate), which is guided and imaged with an optical system at the quartz substrate–TP layer interface, and as a result of the rapid increase in pressure at the quartz–TP interface, a part of the donor layer (SWCNT or SWCNT@SnO_2_) is transferred (further named pixel) onto the receiving substrate. A computer-controlled *xyz* translation stage allowed the displacement of both donor and receiving substrates with respect to the laser beam. The donor and the receiving substrates were kept in contact. All experiments were carried out under ambient pressure at temperatures close to room temperature. 

As receiver substrates, we used both glass coverslips cut into 25 × 25 × 1 mm^3^ pieces for the post-characterization of the transferred pixels, as well as interdigitated (IDT) electrodes. The IDT electrodes have a similar structure to the commercial sensors (Microsens gas sensor, MSGS 3000). Briefly, the IDT electrodes were fabricated by sputtering a 20 nm chromium layer, followed by a 100 nm platinum layer on top onto a borax glass substrate [5]. 

In order to obtain a stable sensor response, the LIFT-printed sensors were subjected to a heating treatment, similar to the commercial sensors. The LIFT-printed sensors were heated for 6 h at 150 °C, followed by 6 h at 100 °C in a stream of 1 L/min of synthetic air (SA) containing 20% O_2_ and 80% N_2_.

The transferred SWCNT and SWCNT@SnO_2_ pixels, as well as the donor films prior to ablation, were investigated by optical microscopy. The images were acquired with an Olympus SZH 10 Research Stereomicroscope, coupled with a Stingray F145C CCD camera.

The distribution of the SWCNT and SWCNT@SnO_2_ prior to and after their deposition by LIFT was investigated by scanning electron microscopy (SEM). The images were obtained from top-view SEM and were acquired with a Zeiss Supra VP55 FE-SEM apparatus operating at a voltage of 5 kV and using an in-lens detector. 

### 2.3. Sensor Testing Setup

The functionality of the LIFT-printed SWCNT and hybrid SWCNT@SnO_2_-based sensors was assessed in a setup that has been described previously in [5]. Briefly, the sensor testing setup consisted of a sensor-testing chamber provided with a constant gas supply, where the sensors were mounted on an alumina block and were contacted electrically by two metal clamps, on the side pressing graphite rode onto the Pt-electrodes reaching a total contact resistance of less than 50 Ω. Graphite rods were needed to prevent the Pt-electrodes to be scratched off [5]. The resistance measurements were acquired by a computer-controlled (LabView) setup using a Keithley 2400 source meter and Keithley 2000 multimeter. The main gas supply was dry N_2_ (both as balance and purging gas) with a standard gas flow of 5 L/min SA. 

In order to test the LIFT-printed SWCNT and hybrid SWCNT@SnO_2_-based sensors for their ability to detect NH_3_, defined concentrations of NH_3_ were mixed in a balloon from Carl Roth and added in small quantities to the main gas stream. This mixture was added with a low flow rate (0.01 to 0.1 L/min) to the main gas flow and analyte concentrations in the ppm range were thus achieved [5].

## 3. Results and Discussion

### 3.1. LIFT Printing

**Figure 2:**
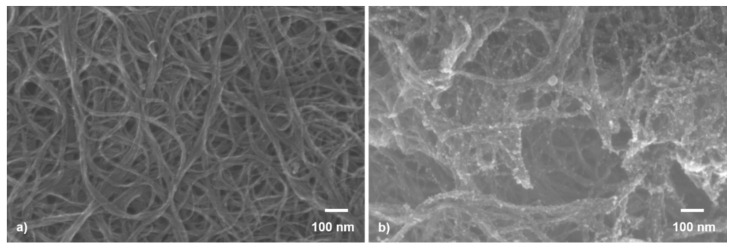
Representative SEM images of (**a**) SWCNT and (**b**) SWCNT@SnO_2_ donors, as fabricated prior to laser transfer.

The gas-sensing mechanism of the sensors is dependent upon multiple factors, including the surface of the active material; therefore, the analysis of the donor films morphology was conducted first. Following our donor fabrication strategy shown in Figure 1, we could spin coat randomly oriented SWCNTs arranged in curly bundles (see Figure 2a). In addition, several μm long carbon nanotube bundles with diameters in the range 30 to 70 nm, and SnO_2_ NP, which agglomerate in clusters, with 10 to 15 nm dimensions, anchored to the SWCNT surface in the hybrid donor materials, which can be seen in the SEM images shown in Figure 2b. The SnO_2_ NPs are regularly distributed on the SWCNT, as shown in Figure 2b. However, in some areas, the SnO_2_ NPs are specifically agglomerated at points where the carbon nanotubes have a close bond, which has also been seen in our previous work [22], where the NPs agglomerated at defect points onto the surface of carbon nanowalls. 

The transfer of pixels in a controlled manner, and insight on the morphological properties of the LIFT-printed single-walled carbon nanotubes and hybrid SWCNT@SnO_2_ are essential in applications aimed at practical devices. Once pixel deposition is possible, and controlled printing and regular pixel deposition can be achieved. In our previous work [16], we have investigated different LIFT process parameters, i.e., the TP thickness and the laser fluence applied for the transfer in order to obtain regular SWCNT patterns. Furthermore, in order to prove that LIFT is a suitable technique to be used for device fabrication, SWCNTs were transferred onto Pt electrodes, and concentrations as low as 50 ppm of ammonia were detected. However, more work is needed to optimize the process and also to obtain better sensor response, i.e., for consistent monitoring of acceptable concentrations of NH_3_. 

**Figure 3:**
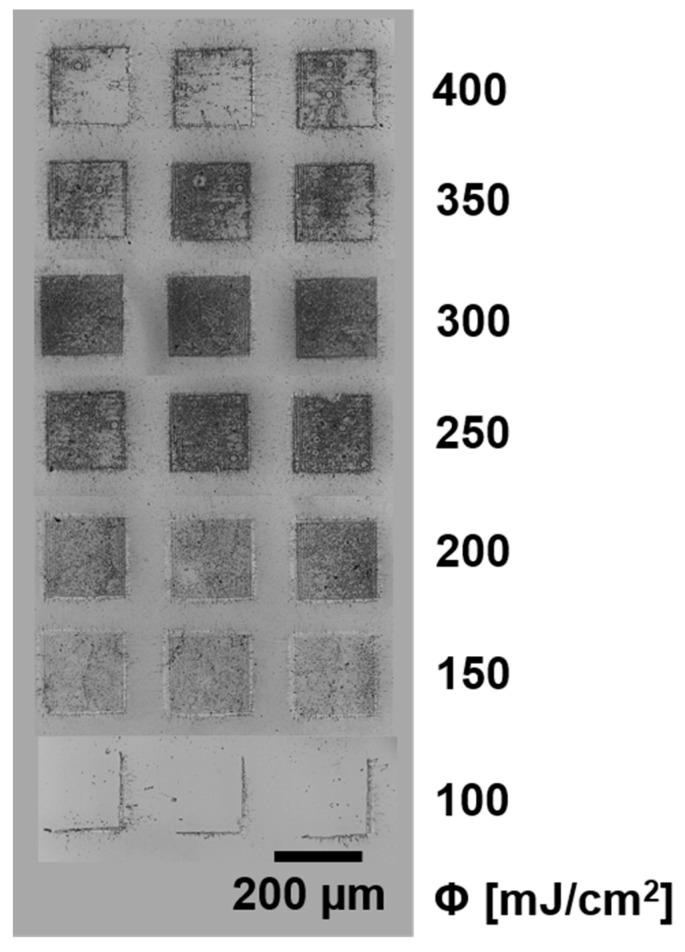
SWCNT@SnO_2_ microarray, transferred by LIFT by keeping the donor and receiver substrates in contact.

Here, we show that it is possible to transfer regular SWCNT@SnO_2_ with the help of a 150 nm thick TP DRL layer (see Figure 3). Transferring SWCNT@SnO_2_ by LIFT is possible, leading to sharp pixels; the best transfer is achieved with a laser fluence of 300 mJ/cm^2^ (similar to the transfer of bare SWCNTs at 250 mJ/cm^2^ in [16]). The overall appearance of the pixel is more “microporous” than homogeneous. Additionally, the transfer fluence of 700 mJ/cm^2^ is comparably high and for lower fluence, no complete layer transfer is achieved. As it can be seen, reproducible transfers with an intermediate 150 nm thick TP layer and a laser fluence of 300 mJ/cm^2^ are achievable.

**Figure 4:**
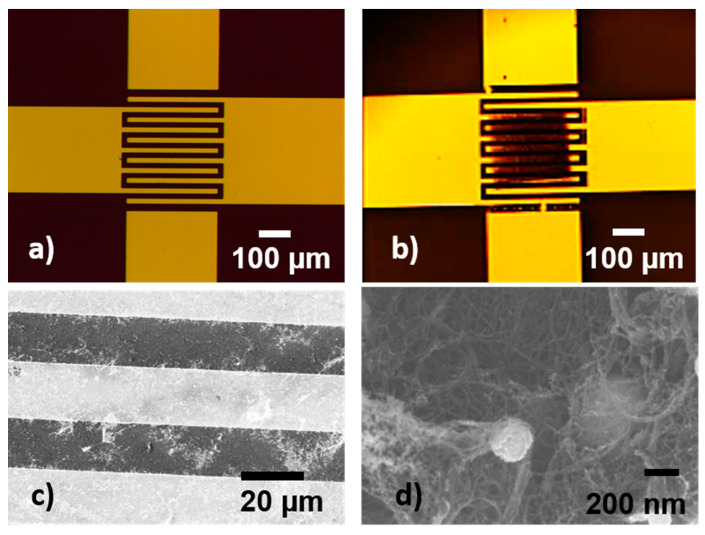
Optical microscopy images of (**a**) the interdigital electrodes and (**b**) a SWCNT@SnO_2_ pixel printed onto the metal electrodes; (**c**) SEM image of SWCNT@SnO_2_ pixel transferred by LIFT at 300 mJ/cm^2^ laser fluence onto the sensor IDTs, and (**d**) SEM image that was taken at a higher magnification of a SWCNT@SnO_2_ pixel transferred by LIFT at 300 mJ/cm^2^ laser fluence onto the sensor IDTs, where the SnO_2_ nanoparticles decorating the SWCNTs can be seen.

In addition, pixels were transferred at 300 mJ/cm^2^ laser fluence onto sensor-like pads, and their electrical characteristics and sensing abilities toward ammonia were tested further. The electrode design and an image of a LIFT-printed sensor-like pad are shown in Figure 4.

The transfers onto the IDT structure do not show significant differences from the transfers onto the glass part. An example of a SWCNT@SnO_2_ pixel transferred onto a sensor-like pad is shown in Figure 4. After the transfer, the as-fabricated sensors were conditioned as described above in order to cure the transferred material. During LIFT, the high velocities generated (around 200 m/s) [16] lead to firm electrical contact between the electrodes and pixels, allowing better draining of the injected carriers through the electrodes. 

These data show that LIFT transfers onto different substrate materials work very well. Transferring onto the IDTs with a structure height comparable to the film thickness shows a similar behavior as the transfers on the even surfaces. Even transfers of SWCNT@SnO_2_ pixels with a film thickness smaller than the IDTs structure height proved to be possible. 

### 3.2. Sensor Tests

The scope of this study is to demonstrate the fabrication of proof-of-concept sensors by directly printing the active materials in the sensors, i.e., the hybrid SWCNT@SnO_2_ nanocomposite via laser-induced forward transfer. Here, we focused on investigating the functionality of the laser-printed active materials onto sensor structures by evaluating their potential to detect small amounts of ammonia at temperatures close to room temperature in dry nitrogen. The fabrication of sensors that operate at a temperature close to room temperature simplifies the sensor design and, in addition, allows for the elimination of the heating elements, which, in turn, offers low-power consumption devices. Furthermore, we chose ammonia, as it represents a well-known toxic pollutant and biological signaling molecule [2].

The first step was to stabilize both the SWCNT and SWCNT@SnO_2_ sensors under N_2_. This was achieved by exposure for 1 h in order to obtain a flat baseline. The baseline resistance (two-point electrical measurement) was measured in a dry N_2_ stream, at room temperature, and for both SWCNT and SWCNT@SnO_2_ sensors, is typically between 2 and 15 kOhms, a resistance range very applicable for sensors and much lower than the Megaohm range found for sensors with standard SWCNT [9]. In addition, during the measurements, the temperature T was continuously measured for the SWCNT and SWCNT@SnO_2_ (T = 25 ± 2 °C). 

**Figure 5:**
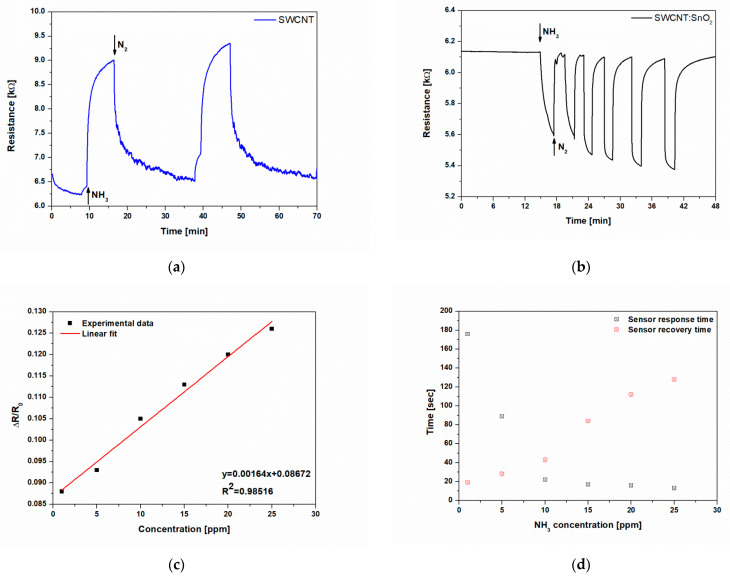
(**a**) Real-time measurements of an SWCNT sensor printed by LIFT collected for 25 ppm of NH_3_; (**b**) real-time measurements of a SWCNT@SnO_2_ sensor printed by LIFT collected for different concentrations (1–25 ppm) of NH_3_; (**c**) SWCNT@SnO_2_ sensor response as a function of NH_3_ concentration at room temperature in air, depicting a good linear relation; (**d**) response and recovery times for the SWCNT@SnO_2_ sensor printed by LIFT exposed to different concentrations of NH_3_ (1–25 ppm).

Real-time measurement of an SWNCT-printed sensor to 25 ppm NH_3_ is shown in Figure 5a. The first observation that can be made is that after exposure to NH_3_, the resistance of the SWCNT sensors is characterized by a steep increase in a short time, and when the analyte is removed, by a slower decrease. This shows a p-type response of the SWCNTs to NH_3_, i.e., after exposure to NH_3_, the resistance of the sensors increases from approx. 6.5 kΩ to 9 kΩ (Figure 5a). This effect has been seen previously [7,9] in SWCNTs exposed to reducing molecules. Briefly, when the surface of the SWCNTs is exposed to the reducing NH_3_ molecules, the accumulation region is reduced, yielding a decrease of the hole current, i.e., an increase of the resistance, shown by the SWCNT sensors printed by LIFT. 

In order to fabricate high-performance sensors, a prerequisite is their high reversibility in response when exposed to multiple cycles of the analyte of interest. The response of SWCNT printed by LIFT is partially reversible at the applied NH_3_ concentration of 25 ppm, demonstrating a quasi-dosimetric characteristic of the sensors.

Although we could have applied different remedies to achieve full reversibility, i.e., heating or UV exposure of the SWCNT sensors, we noticed that these treatments led to a high baseline noise, and therefore, we looked into the direction of noncovalent SWCNT functionalization by decorating the SWCNTs with metal oxide nanoparticles (SnO_2_). In particular, the strategy of introducing active sites with strong affinities has been followed previously in order to improve sensor sensitivity [23,24]. 

In addition, at first, the decoration of the SWCNTs with n-type NPs might appear striking, due to the fact that the two opposing mechanisms may cancel each other, thus diminishing the sensor performance. In order to evaluate the SWCNT@SnO_2_ sensors fabricated by LIFT for their ability to detect NH_3_ molecules, we tested them against different concentrations, i.e., in the range 1–25 ppm. We noticed that in contrast to the laser-printed SWCNT sensors, the resistance of the SWCNT@SnO_2_ sensor after exposure to NH_3_ immediately decreases and gradually approaches a steady-state over a period of ~2 min, thus showing an n-type response to NH_3_. Therefore, it is safe to assume that the overall characteristic of the SWCNT@SnO_2_ sensors is determined by the properties of the SnO_2_ NPs, and the SWCNTs contribute little to the sensor response. This claim is supported also by the SEM image shown in Figure 2b, in which it can be seen that most of the SnO_2_ NPs are in contact with each other. 

Although the exact mechanism of the hybrid sensor response is not known, we hypothesize that the better sensor response toward NH_3_ analyte of the SWCNT@SnO_2_ sensors, as compared with the SWCNT sensors, could be attributed to a number of factors, i.e., the enhancement of the surface area accessible to the ammonia molecules, and possibly to the formation of p–n junctions between the semiconducting metal oxide NP and the CNT bundles. In the literature, there are many studies focused on the gas sensing mechanism in n-type semiconducting metal oxide nanoparticles and p-type carbon nanotubes, where these materials are combined to form p–n junctions. The case of p–n junctions between p-type SWCNTs and n-type semiconductor NP presented on hybrid SWCNT@NPs [25], however, a clear understanding of the mechanisms leading to gas sensing has not yet been reached [26]. 

Additional experimental and theoretical studies are needed to fully understand the interaction of the hybrid SWCNT@SnO_2_ system with ammonia, to identify the hybrid sensor response as a function of the applied temperature, as well as to determine the dominant material for different SnO_2_ NP coverage.

In addition, the SWCNT@SnO_2_ sensor response is reproducible over multiple analyte/N_2_ exposure cycles, and, more importantly, the signal recovered to the original baseline value upon removal of the NH_3_ (Figure 5b), thus demonstrating a sensor behavior.

In order to evaluate the performance of our printed sensors, we investigated several key factors: (i) the sensor response to ammonia, which is defined as ΔR/R_0_ = (R_g_ − R_0_)/R_0_, where R_g_ is the resistance upon NH_3_ exposure, and R_0_ the baseline resistance before exposure to NH_3_; (ii) the response and recovery times, which are defined as the times for the sensor to reach 90% of its maximum and to recover 10% of its peak value upon exposure to a given concentration of NH_3_. 

Although both SWCNT and SWCNT@SnO_2_ sensors display a resistivity change, both the response and recovery appear to be much slower in the case of SWCNT with respect to the hybrid nanocomposite SWCNT@SnO_2_ sensors. In order to obtain quantitative correlations between the changes in the sensor responses as a function of NH_3_ concentration, we carried out concentration-dependent investigations. An excellent linear sensor response as a function of the tested NH_3_ concentrations (1–25 ppm) is depicted in Figure 5c, with R^2^ = 0.98. 

Furthermore, due to the limitations of our experimental setup, concentrations as low as 1 ppm could be detected in dry nitrogen without any external aid such as thermal or photoirradiation (see Figure 5b). Therefore, we calculated the theoretical detection limit (LOD) (additional information can be found in Supplementary Information), as previously reported [11], from the signal/noise ratio. 

The slope (0.00164) was obtained from the calibration curve of the sensor response in Figure 5c. For the LIFT-printed SWCNT@SnO_2_ sensors, we obtained a LOD = 23.93 ppt, which is among the lowest LOD value reported by other chemiresistive sensors based on hybrid carbon nanotubes and tin oxide nanoparticles [27]. Even more, this LOD is two orders of magnitude lower than the LOD of a single tin oxide nanowire [28].

For 1 ppm of ammonia, the response and recovery times of the SWCNT@SnO_2_ sensor are 176 s and 19 s, respectively (see Figure 5d). These values are among the best reported in the literature (see Table 1). By increasing ammonia concentration, i.e., to 25 ppm, the response time decreases to 13 s, while the recovery time increases moderately to 123 s, which is still better than most in the literature (see Table 1). 

A brief evaluation of the recovery and response times for the different types of sensors evaluated in this work and a comparison with other types of sensors that are exposed to different concentrations of ammonia are shown in Table 1. The results we present in this work show that the printed hybrid SWCNT@SnO_2_ are promising candidates for the fast detection of NH_3_ at room temperature.

Therefore, the approach presented here, combining special materials in the form of carbon nanotube-based nanocomposites and the designed polymer absorbers for the laser-based printing, combined with an advanced laser direct-write approach is very attractive to advance the state-of-the-art in sensing devices. 

## 4. Conclusions

In summary, we successfully demonstrated the solvent-free fabrication of SWCNT@SnO_2_ nanocomposite-based sensors. The hybrid SWCNT@SnO_2_ nanocomposites were laser printed with high resolution onto specific metallic geometries designed onto glass substrates. The resulting sensors are reproducible and were tested against different concentrations of NH_3_. Upon NH_3_ testing (at room temperature), the SWCNT@SnO_2_ sensors exhibit a fast and reversible response over multiple cycles and have a theoretical detection limit in the low ppt range (i.e., 24 ppt). In perspective, this study provides an opportunity to fabricate sensors by a simple technique, compatible with a scale-up process, for monitoring sub-ppm ammonia concentrations. The fast response and recovery times, together with the low detection limit required for realistic monitoring of ammonia concentrations, are achieved. Thus, the implementation of SWCNT@SnO_2_ laser-printed sensors that can provide reliable monitoring of NH_3_ represent the basis for future advanced sensing devices.

## Figures and Tables

**Table 1:** Summarized sensor parameters for different works, as compared with the SWNT@SnO_2_ sensors fabricated by LIFT.

Sensing Material	Operating Temperature	Concentration (ppm)	Sensor Response (%)	Response Time	Recovery Time	Ref.
SWCNT@SnO_2_	RT	25	0.126	13 s	123 s	This work
SnO_2_ + 15%MWCNT	RT	200	0.27	<5 min	<5 min	[29]
SnO_X_-SWNT	200 °C	1000	0.81	2.02 min	3.14 min	[30]
SnO_2_ + 1%wt MWCNT	220	60	0.19	>100 s	>100 s	[31]
Cellulose fiber	RT	0.2–1000	40 (80 ppm)	186 s	163 s	[32]
SnO_2_-Pt	230 °C	1	-	1 s	59 s	[33]
Co_3_O_4_ nanosheets	RT	0.2–100	-	9 s	134 s	[34]
TiO_2_/Ti_3_C_2_T_x_	RT	0.5–10	3.1 (10 ppm)	33 s	277 s	[35]
CuBr	RT	5 ppb–5 ppm	800 (500 ppb)	132 s	50 s	[36]

## Data Availability

The data used to support the findings of this study are available from the corresponding author upon request.

## Supplementary Materials

The following are available online at https://www.mdpi.com/article/10.3390/nano11102604/s1, Figure S1: The calculation of the theoretical detection limit.
