# Supplementary material for: Facile Fabrication of Hybrid Carbon Nanotube Sensors by Laser Direct Transfer
Source: Nanomaterials (Basel). 2021 Oct 3;11(10):2604. doi: 10.3390/nano11102604 (PMC8539917; doi:10.3390/nano11102604)
Supplement: Supplementary file 1 [file nanomaterials-11-02604-s001.zip › nanomaterials-1399482-supplementary.pdf]

nanomaterials

Supplementary Materials

Facile Fabrication of Hybrid Carbon Nanotube Sensors by Laser Direct Transfer

Anca F. Bonciu [1,2], Mihaela Filipescu [1], Stefan I. Voicu [3,4], Thomas Lippert [5,6], Alexandra Palla-Papavlu [1,*]

[1] National Institute for Lasers, Plasma and Radiation Physics, Lasers Department, Atomistilor 409, 077125, Magurele, Romania;
[2] Faculty of Physics, University of Bucharest, Atomistilor 409, 077125, Magurele, Romania
[3] University Politechnica of Bucharest, Department of Analytical and Environmental Chemistry, 1-7 Gh. Polizu Str., 011061, Bucharest, Romania
[4] Advanced Polymer Materials Group, University Politehnica of Bucharest, 1-7 Gh. Polizu Str., 011061, Bucharest, Romania
[5] Laboratory of Inorganic Chemistry, Department of Chemistry and Applied Biosciences, ETH Zurich, 8093 Zurich, Switzerland
[6] Laboratory of Multiscale Materials Experiments, Paul Scherrer Institute, 5232 Villigen, Switzerland
* Correspondence: Alexandra.papavlu@inflpr.ro

We have calculated the theoretical detection limit (LOD) as previously reported [1] from the signal/noise ratio. First, we took 13 data points at the baseline before NH_3 exposure (y_i) and plotted the data using a fifth order polynomial fit.

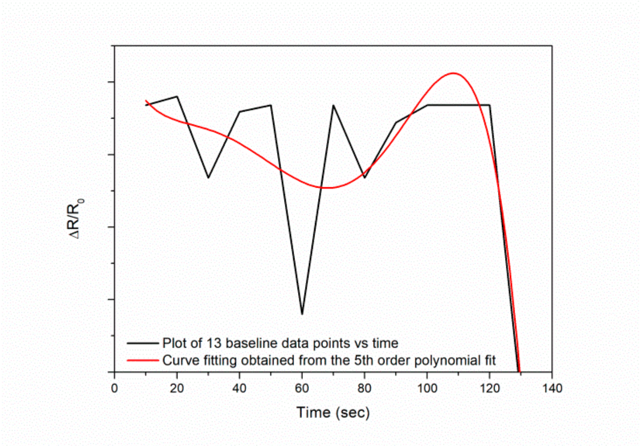

Figure S1. Calculation of the theoretical detection limit.

From the curve-fitting equation we obtained the statistical parameters of the polynomial fit (y), and then calculated the sensor noise using the root-mean-square deviation (Eq. 1).

Eq. (1) $rms_{noise}=\sqrt{\Sigma(y_i-y)^2}/N$

$\sqrt{\Sigma(y_i-y)^2}=1.23639E-08$

N=13, where N is the number of data points in the curve fitting.

The rms_{noise} for the SWCNT@SnO_2 sensor is 7.09599E-5.

The theoretical detection limit according to IUPAC is

Citation: Bonciu, A.F.; Filipescu, M.; Voicu, S.I.; Lippert, T.; Palla-Papavlu, A. Facile Fabrication of Hybrid Carbon Nanotube Sensors by Laser Direct Transfer. *Nanomaterials* **2021**, *11*, 2604. https://doi.org/10.3390/nano11102604

Academic Editor: Johannes Heitz

Received: 13 September 2021
Accepted: 1 October 2021
Published: 3 October 2021

Publisher's Note: MDPI stays neutral with regard to jurisdictional claims in published maps and institutional affiliations.

Copyright: © 2021 by the authors. Licensee MDPI, Basel, Switzerland. This article is an open access article distributed under the terms and conditions of the Creative Commons Attribution (CC BY) license (http://creativecommons.org/licenses/by/4.0/).

Eq. (2) $LOD=3\times rms_{noise} / slope$.

The slope is 0.00164 (obtained from the calibration curve of the sensor response)

LOD = 23.93 ppt

References

1. Ammu, S.; Dua, V.; Agnihotra, S.R.; Surwade, S.P.; Phulgirkar, A.; Patel, S.; Manohar, S.K. Flexible, All-Organic Chemiresistor for Detecting Chemically Aggressive Vapors. *J. Am. Chem. Soc.* **2012**, *134*, 4553–4556, doi:10.1021/ja300420t.
